# Comment on ‘integrating systemic inflammation and liver biomarkers: prognostic implications of the ferritin index in heart failure’

**DOI:** 10.1080/07853890.2025.2560037

**Published:** 2025-09-16

**Authors:** Xiaobo Shen, Bingyu Lin, Tianchen Lin

**Affiliations:** ^a^ Huzhou Central Hospital, Fifth School of Clinical Medicine of Zhejiang Chinese Medical University, Huzhou, Zhejiang, China; ^b^ Hangzhou Medical College, Hangzhou, Zhejiang, China; ^c^ Affiliated Hangzhou First People’s Hospital, School Of Medicine, Westlake University, Hangzhou, Zhejiang, China

To the Editor

We read with interest the article by Huang et al. entitled ‘Integrating systemic inflammation and liver biomarkers: prognostic implications of the ferritin index in heart failure’ [1]. The authors should be commended for exploring the role of the ferritin index in predicting major adverse cardiovascular events (MACE) in patients with heart failure (HF), particularly in the context of metabolic hyperferritinemia (MHF). However, several methodological and conceptual limitations merit further discussion.

The discriminative performance of the ferritin index was modest (AUC = 0.560), suggesting limited clinical utility as a standalone biomarker. Combining it with established predictors like NT-proBNP or troponin may enhance prognostic accuracy, consistent with multi-marker strategies.

A significant selection bias arises from excluding 77% of the initial cohort due to missing ferritin data. This reflects the real-world underutilization of iron studies. Although acknowledged by the authors, this issue still limits the representativeness and external validity of the findings.

The inclusion of new-onset atrial fibrillation (AF) in the composite endpoint of major adverse cardiovascular events (MACE) is debatable. While AF is increasingly recognized as a clinically relevant complication in HF, its heterogeneous mechanisms and prognostic implications differ from those of hard endpoints such as myocardial infarction or stroke [2]. A stratified analysis or separate reporting would strengthen the conclusions.

In addition, a limitation is the lack of longitudinal ferritin measurements, which may better reflect chronic inflammatory status. Ferritin is influenced by acute-phase responses, and a single baseline measurement may not adequately reflect chronic inflammatory or iron status. Serial measurements could provide more robust prognostic information, as suggested in prior HF cohorts [3].

Moreover, while the ferritin index incorporates age and sex adjustments, it does not distinguish between inflammation-driven ferritin elevation and true iron overload. Complementary iron parameters such as transferrin saturation or soluble transferrin receptor could help clarify the underlying pathophysiology and improve risk stratification [4]. Given MHF’s overlap with acute-phase responses, subgroup analyses stratified by C-reactive protein levels would strengthen causality.

Finally, the retrospective single-center design inherently restricts generalizability. Although inverse probability weighting (IPTW) was applied to mitigate confounding, unmeasured variables, such as dietary iron intake, occult inflammatory conditions, or differences in clinical management, remain possible sources of bias. Prospective multi-center studies are needed to validate these findings in the future.

In summary, while the study introduces the ferritin index as an innovative marker for HF prognosis, its modest predictive ability, potential selection bias, and endpoint definition warrant cautious interpretation. Future multicenter prospective studies incorporating multi-marker panels are essential before clinical adoption.

## Data Availability

Data sharing is not applicable to this article as no new data were created or analyzed in this study.

## References

[1] Huang CH, Kor CT, Chang CC. Integrating systemic inflammation and liver biomarkers: prognostic implications of the ferritin index in heart failure. Ann Med. 2025;57(1):2540020. doi: 10.1080/07853890.2025.2540020. 40746303 PMC12320259

[2] Linz D, Gawalko M, Betz K, et al. Atrial fibrillation: epidemiology, screening and digital health. Lancet Reg Health Eur. 2024;37:100786. doi: 10.1016/j.lanepe.2023.100786. 38362546 PMC10866942

[3] Karakas M, Friede T, Butler J, et al. Intravenous ferric carboxymaltose in heart failure with iron deficiency (FAIR-HF2 DZHK05 trial): sex-specific outcomes. Eur J Heart Fail. 2025; Published online July 31, doi: 10.1002/ejhf.3742. PMC12765045 40740027

[4] Zhang Z, Wu P, Yang S, et al. The association between frailty index and abdominal aortic calcification in the middle-aged and older US adults: NHANES 2013–2014. Front Public Health. 2025;13:1546647. doi: 10.3389/fpubh.2025.1546647. 40401062 PMC12092342

